# Effects of virtual reality and nature sounds on pain and anxiety during hysterosalpingography: a randomized controlled trial

**DOI:** 10.1590/1806-9282.20231599

**Published:** 2024-08-16

**Authors:** Nazlı Baltaci, Sümeyye Bal, Emine Koç, Elif Keten Edis

**Affiliations:** 1 Ondokuz Mayıs University, Faculty of Health Sciences, Department of Nursing - Samsun, Turkey.; 2 Ondokuz Mayıs University, Faculty of Health Sciences, Department of Midwifery - Samsun, Turkey.; 3 Amasya University, Faculty of Health Sciences, Department of Nursing - Amasya, Turkey.

**Keywords:** Anxiety, Hysterosalpingography, Nature, Pain, Virtual reality

## Abstract

**OBJECTIVE:**

The objective of this study was to determine the effects of listening to nature sounds alone and virtual reality plus listening to nature sounds on pain and anxiety in hysterosalpingography.

**METHODS:**

This three-arm parallel randomized controlled trial included 135 (45 in each group) women who underwent hysterosalpingography in Turkey. The virtual reality+nature sounds group viewed a nature video with virtual reality glasses and listened to nature sounds during hysterosalpingography, whereas the nature sounds group only listened to nature sounds. The control group received only routine care.

**RESULTS:**

During hysterosalpingography, women in virtual reality+nature sounds group experienced less pain than those in control group (p=0.009). After hysterosalpingography, pain levels were lower in both virtual reality+nature sounds group and nature sounds group than in control group (p=0.000 and p=0.000, respectively), anxiety levels were lower in virtual reality+nature sounds group than in nature sounds group and control group (p=0.018 and p=0.000, respectively), and anxiety levels were lower in nature sounds group than in control group (p=0.013).

**CONCLUSION:**

Virtual reality with nature content plus listening to nature sounds and only listening to nature sounds are effective in reducing pain and anxiety related to hysterosalpingography procedures in women. Compared with only listening to nature sounds, virtual reality plus listening to nature sounds further reduced hysterosalpingography-related pain and anxiety.

## INTRODUCTION

Hysterosalpingography (HSG) is an uncomfortable and invasive procedure associated with fear, anxiety, and pain^1^. HSG is the radiological evaluation of the tuba uterinas, ovaries, and uterus by transcervical administration of contrast material in the investigation of the causes of infertility. This invasive and risky method is widely used for diagnosis and treatment with the help of instruments inserted into the uterus^2,3^. Various studies have reported that women experience moderate or severe pain during the HSG procedure, with an incidence of 59.3-93.7%^3–5^. It is known that women experience anxiety about the uncertainty before undergoing HSG and the possible pain, which can negatively affect the procedure, and are reluctant to undergo the procedure^1^.

Different pharmacological agents and applications have been used to reduce pain among women during HSG^6^. However, to date, there is no ideal method that can be recommended to reduce pain during HSG. Some randomized controlled trials on integrated medicine have reported that during HSG, primrose and transabdominal pelvic hot water bag applications can reduce pain^7,8^ and acupuncture can reduce anxiety^4^.

Virtual reality (VR) is one of the recent important technological developments that can be used as a noninvasive and effective analgesic method during painful invasive procedures^9^. As a relaxation technique, VR can suppress adrenaline secretion and increase endorphin and oxytocin secretion. It can reduce pain by regulating mood and hormonal effects by diverting attention^10^. VR is used in women's health, such as colposcopy, cystoscopy, psychological problems^11^, hysteroscopy^12^, cesarean section^13^, and labor pain^14^.

The physical and mental unity of individuals with nature is closely related to their health. It has been reported that watching nature accelerates the healing process and nature calms individuals by diverting attention^15^. Nature sounds (NS) are one of the interventions used to reduce anxiety in recent years. The sounds of the sea, rain, wind, river, birds, and other animals in nature can increase parasympathetic activity and reveal physiological and psychological responses^15,16^. NS have been reported to reduce pain after cesarean section^17^ and provide psychological and physiological relaxation^16^.

To the best of our knowledge, while there are only two studies on the effectiveness of VR in women undergoing HSG^1,18^, there is no study on the effectiveness of NS. Furthermore, no studies have investigated the effects of using VR with nature content plus listening to NS on pain and anxiety in women before and during HSG. Furthermore, it has been stated that there is a need for studies that investigate the effects of VR accompanied by music on pain and anxiety before and during HSG^10^. Therefore, the study aimed to determine the effects of listening to NS alone and VR plus listening to NS on pain and anxiety in women before and during HSG.

## METHODS

### Research type

This was a three-arm parallel randomized controlled trial with a pretest-posttest experimental design. The study was conducted in accordance with the CONSORT guidelines and was registered on ClinicalTrials.gov (registration number: NCT05192343).

### Setting and sample

The women who wanted to participate in the study were recruited from the radiology center of a university hospital in northern Turkey between February and August 2022. Inclusion criteria were as follows: women who underwent HSG, those aged>18 years, and those who could read and speak Turkish. Exclusion criteria were as follows: women with psychological and mental health problems and those with hearing or visual impairments.

The sample size was calculated with a 95% confidence level (1 - α), 95% test power (1 - β), and an effect size of 0.804 (d) by performing power analysis according to the pain level reported in a previous study^4^. Accordingly, the minimum sample size was calculated as 36 women in each group. Considering possible data loss, this study included a total of 135 women, with 45 in each group.

### Randomization and blinding

**Figure 1:**
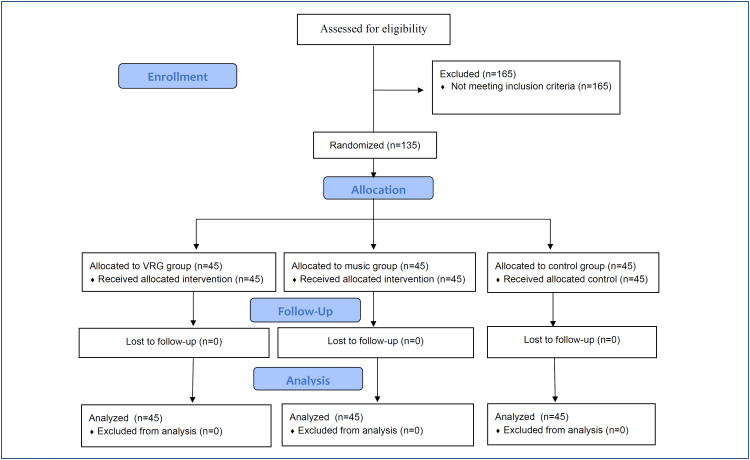
CONSORT flow diagram.

Randomization into one of the three study groups was performed by a statistician blinded to the participants using the Quickcalcs GraphPad program (http://www.graphpad.com/quickcalcs/, accessed on January 20, 2022) (Figure 1). Owing to the nature of the biobehavioral interventions, participants and researchers could not be blinded.

### Interventions

The women in VR plus listening to NS group (VR+NSG) viewed a relaxing video with nature content by listening to the sounds of nature with 360° VR glasses for 30 min and 15 min before and during the HSG procedure, respectively. The use of VR glasses was explained to the women before the procedure. The women in the listening to NS group (NSG) only listened to a recording of NS for 30 min and 15 min before and during the HSG procedure, respectively. The recording included sounds of water, waves, rain, wind, dolphins, birds, and forests from Savva's (2019) CD entitled "NS for Babies" and from the CD entitled "Nature Symphonies" (2007). As suggested in the relevant literature^19^, NS used in this study were relaxing, with a soft melody, a slow rhythm (ranging between 60 and 70 beats per minute, as measured using a metronome), and consistent with the normal heart rate. Before performing the HSG procedure, NS were played in a quiet room with an MP3 music player and disposable earbuds to prevent the women from being affected by ambient sounds. The control group (CG) only received routine care.

### Data collection

The data were collected with the "Women Information Form" developed by the researchers in line with the relevant literature^5,7,17^, "Visual Analog Scale (VAS)," and "Spielberger State-Trait Anxiety Inventory (STAI)."

### Visual Analog Scale

Visual Analog Scale is a one-dimensional 0-10-cm-long scale developed by Hayes and Patterson in 1921, which is commonly used to measure pain intensity. The scale parameters range from "no pain (0)" to "intolerable pain (4)." Higher scores indicate greater pain severity^20^. In this study, pain was evaluated during and 15 min after the HSG procedure.

### State-Trait Anxiety Inventory

State-Trait Anxiety Inventory was developed by Spielberger et al.^21^, and the Turkish validity and reliability study of this inventory was conducted by Öner and Le Compte^22^. It includes two self-report scales: a 4-point Likert type and a 20-item state and trait anxiety inventory. Scores between 20 and 80 can be obtained from both scales. The higher the score obtained from the scales, the higher the level of anxiety. The Cronbach's alpha reliability coefficients were 0.83-0.92^22^. In this study, anxiety was evaluated immediately before and 15 min after the HSG procedure.

### Ethics

This study was approved by the Clinical Research Ethics Committee of Ondokuz Mayıs University (decision dated 30.09.2021, numbered 2021/435). The study was conducted on a voluntary basis, and written informed consent of the women was obtained before the study.

### Data analysis

In the IBM SPSS Statistics Version 23.0 program, analysis of variance (ANOVA) and Tukey test were used for the comparison of VAS and STAI scores between groups, while paired-sample t-test was used for the comparison within groups. Statistical significance was indicated by p<0.05.

## RESULTS

In this study, between February and August 2022, 300 participants were assessed for eligibility, 165 of whom were excluded. Finally, 135 participants were included and randomly assigned to VR+NSG (n=45), NSG (n=45), and CG (n=45). After randomization, none of the participants discontinued intervention. Overall, 45 participants in each group completed the study and were analyzed (Figure 1).

Table 1 shows that the groups were similar, and there was no significant difference between the groups in terms of their age, duration of marriage, educational and employment status, body mass index, duration of menstruation, menstrual pattern, dysmenorrhea, having children, undergoing HSG for the first time, and receiving HSG information (p>0.05) (Table 1).

**Table 1:** Descriptive characteristics of women.

Variables		Virtual reality+nature sounds group (n=45)	Nature sounds group (n=45)	Control group (n=45)	p-value
Age (years)		29.55 (6.09)	30.11 (5.99)	28.60 (4.15)	0.420^a^
Duration of marriage (years)		5.13 (5.19)	3.24 (2.14)	4.13 (2.89)	0.053^a^
Education status					
	Secondary school	10 (22.2)	4 (8.9)	5 (11.1)	
	High school	9 (20.0)	9 (20.0)	7 (15.6)	0.339^b^
	University	26 (57.8)	32 (71.1)	33 (73.3)	
Employment status					
	Employed	17 (37.8)	22 (48.9)	18 (40.0)	0.529^b^
	Unemployed	28 (62.2)	23 (51.1)	27 (60.0)	
Body mass index					
	Underweight	2 (4.4)	1 (2.2)	1 (2.2)	
	Normal	22 (48.9)	19 (42.2)	23 (51.1)	0.931^b^
	Overweight	9 (20.0)	13 (28.9)	11 (24.4)	
	Obese and morbidly obese	12 (26.7)	12 (26.7)	10 (22.2)	
	Duration of menstruation (days)	6.04 (1.67)	5.55 (1.82)	6.17(1.89)	0.230^a^
Menstruation pattern					
	Regular	36 (80.0)	38 (84.4)	33 (73.3)	0.425^b^
	Irregular	9 (20.0)	7 (15.6)	12 (26.7)	
Dysmenorrhea					
	Yes	28 (62.2)	34 (75.6)	26 (57.8)	0.183^b^
	No	17 (37.8)	11 (24.4)	19 (42.2)	
Having children					
	Yes	11 (24.4)	8 (17.8)	13 (28.9)	0.459^b^
	No	34 (75.6)	37 (82.2)	32 (71.1)	
Undergoing HSG for the first time					
	Yes	32 (71.1)	40 (88.9)	33 (73.3)	0.087^b^
	No	13 (28.9)	5 (11.1)	12 (26.7)	
Receiving hysterosalpingography information					
	Yes	31 (68.9)	32 (71.1)	29 (64.4)	0.788^b^
	No	14 (31.1)	13 (28.9)	16 (35.6)	

Categorical variables are presented as n (%) and continuous variables are presented as mean (SD).

a One-way analysis of variance (ANOVA);

b chi-square test.

A significant difference was found between the groups in terms of pain levels during the HSG procedure (p=0.013). There was no difference between the pain levels of VR+NSG and NSG during the procedure (p>0.05), but the pain level of VR+NSG was significantly lower than that of CG (p=0.009). Moreover, a significant difference was found between the groups in terms of pain levels after the HSG procedure (p=0.000). After the procedure, the pain levels of both VR+NSG and NSG were significantly lower than those of CG (p=0.000 and p=0.000, respectively). Regarding within-group comparisons, there was a significant difference in the VAS scores of the intervention groups (p=0.000) (Table 2).

**Table 2:** Pain and anxiety levels of women according to groups and measurement times.

VAS		Virtual reality+nature sounds group(n=45)	Nature sounds group(n=45)	Control group(n=45)	Between groups p-value^a^
During HSG		8.15 (2.28) x	8.71 (1.96) y	9.33 (1.16) z	0.013
After HSG		3.62 (1.89) x	3.46 (2.71) y	9.26 (3.03) z	0.000
Within group p-value^b^		0.000	0.000	0.183	
STAI					
	Pre-HSG	90.37(12.88) x	87.77 (8.86) y	87.24 (6.95) z	0.277
	Post-HSG	85.42 (8.07) x	90.02 (9.51) y	94.80 (5.64) z	0.000
	Within group p-value^b^	0.004	0.125	0.000	

Data are presented as mean (SD).

a One-way analysis of variance (ANOVA);

b paired-sample t-test; x, y, and z: according to the Tukey test, there is no difference between data indicated by the same letter.

A significant difference was found between the groups after the procedure (p=0.000). Anxiety levels were significantly lower in VR+NSG than in NSG and CG (p=0.018 and p=0.000, respectively). Similarly, anxiety levels were significantly lower in NSG than in CG (p=0.013). Regarding within-group comparisons, there was a significant difference in the STAI scores after the procedure in VR+NSG (p=0.004) (Table 2).

## DISCUSSION

Hysterosalpingography is an invasive procedure that can cause anxiety and pain among women. In this study, although the pain levels in the intervention groups were similar during and after HSG, the pain level in VR+NSG was significantly lower than that in CG. In addition, the pain level decreased significantly in VR+NSG after the procedure. It has been mentioned that seeing nature views or spending time in nature can reduce anxiety or negative physical reactions^15^. It is known that the use of VR reduces pain levels and analgesic use during invasive procedures in various patient groups^9,14,23^. Similar to our result, in two randomized controlled studies, women in the VR group had lower pain levels 15 min after HSG than in the CG^1,18^. Similarly, in this study, the use of VR plus listening to NS was effective in reducing pain during and after the injection of a contrast agent into the uterus—the most painful step in HSG.

Anxiety caused by uncertainty related to invasive procedures occurs during HSG and can increase pain perception^1^. In this study, in addition to the pain level, the anxiety level in VR+NSG significantly decreased after the procedure compared with the anxiety level before the procedure and the level in CG. VR can virtually remove an individual from the medical environment and reduce negative emotions by shifting the focus to pleasant stimuli^10^. Some studies on women's health have used VR to reduce anxiety^12,13,23,24^. Different from these studies, in the randomized controlled study of Sezer et al.^1^ the anxiety level of the VR group was not different from the CG during HSG. Furthermore, in this study, anxiety levels after HSG were found to be lower in VR+NSG than in NSG. This finding may be attributed to the following reasons: individuals in VR+NSG were able to focus on the sounds and images of nature; individuals were visually and spiritually removed from the foreign medical environment; and individuals could relax while using images and sounds of nature, which simultaneously affected different senses.

Nature sounds have an intercultural, pleasing, and preferred structure. Listening to NS is a physically and mentally relaxing experience^16^. In this study, we found that the postprocedure level of pain in NSG was lower than the preprocedure level and the level in CG. Similarly, some studies have reported that natural sounds are effective in reducing pain in patients^16,17^. In this study, postprocedure anxiety levels of NSG were higher than those of VR+NSG and lower than those of CG. Studies have found that NS reduce stress and provide psychological relaxation^15,16,25^. In this study, women experienced less anxiety and pain when listening to NS before and during HSG, which may be attributed to the relaxing effects of NS.

This study has two limitations. First, the findings of this study were limited to women who underwent the procedure at a single center. Second, the natural sounds used in the study were preselected by the researcher.

## CONCLUSION

Virtual reality with nature content plus listening to NS and only listening to NS are effective in reducing pain and anxiety related to HSG procedures in women. Compared with only listening to NS, VR plus listening to NS further reduced HSG-related pain and anxiety. This method is an effective, easy-to-apply, noninvasive, and nonpharmacological method to reduce pain and anxiety in women undergoing HSG. The effectiveness of the combined use of VR and NS on pain and anxiety in different patient populations and invasive procedures can be investigated.

## ETHICAL APPROVAL

This study adhered to the principles of the Declaration of Helsinki. This study was approved by the Clinical Research Ethics Committee of Ondokuz Mayıs University (dated 30.09.2021, numbered 2021/435). The study was conducted on a voluntary basis, and written informed consent of pregnant women was obtained before the study.
